# Nebivolol therapy improves QTc and QTcd parameters in heart failure patients

**DOI:** 10.5830/CVJA-2011-046

**Published:** 2012-05

**Authors:** SM Aksoy, S Cay, N Sen, G Cagirci

**Affiliations:** Department of Anesthesiology, Diskapi Yildirim Beyazit Education and Research Hospital, Ankara, Turkey; Department of Cardiology, Yuksek Ihtisas Heart Education and Research Hospital, Ankara, Turkey; Department of Cardiology, Yuksek Ihtisas Heart Education and Research Hospital, Ankara, Turkey; Department of Cardiology, Diskapi Yildirim Beyazit Education and Research Hospital, Ankara, Turkey

**Keywords:** improvement, nebivolol, QT dynamics

## Abstract

**Aim:**

It has previously been shown that β-blocker therapy reduces QT dynamics in heart failure patients. The aim of this study was to demonstrate this improvement with the third-generation β-blocker, nebivolol.

**Methods:**

A total of 72 heart failure patients with systolic dysfunction were included in the study. Corrected QT (QTc) and QT dispersion (QTcd) were measured manually by two independent observers at baseline and after nebivolol use (5 mg/day) in the first and third months of follow up.

**Results:**

Both QTc and QTcd were found to be significantly reduced in the first (455.3 ± 26.7 vs 441.2 ± 25.7 ms, *p* < 0.001 for QTc, and 65.6 ± 5.3 vs 58.2 ± 5.6 ms, *p* = 0.001 for QTcd) and third months (455.3 ± 26.7 vs 436.0 ± 28.7 ms, *p* < 0.001 for QTc, and 65.6 ± 5.3 vs 56.0 ± 6.2 ms, *p* < 0.001 for QTcd) compared with baseline values.

**Conclusion:**

Nebivolol was associated with improved QT dynamics in heart failure patients with systolic dysfunction.

## Abstract

Heart failure, both systolic and diastolic, is a clinical syndrome with different treatment modalities and a poor prognosis, especially in the advanced stages. β-blocker therapy, including nebivolol, improves survival in these patients by modulating several neurohormonal mechanisms.^1^-^4^

Nebivolol is a selective β_1_-blocker with vasodilatory properties due to modulation of nitric oxide release, which decreases peripheral vascular resistance.^5^,^6^ In addition, it has been demonstrated that in patients with hypertension, nebivolol reduces QT dispersion and corrected QT interval (QTc) and corrected QT dispersion (QTcd), which are indicators of the heterogeneity of myocardial repolarisation and electrical instability.^7^ Abnormality of these parameters has been also found to be associated with adverse cardiac events and mortality.^8^

In the current study, we aimed to demonstrate the beneficial effect of nebivolol on QTc and QTcd in patients with heart failure with systolic dysfunction.

## Methods

Consecutive β-blocker-naïve (no use during the four weeks before study entry) systolic heart failure patients were selected for the study. A total of 75 patients were possible candidates and were included in the study. However, three were subsequently excluded because of side effects of the drug. At the end of the follow-up period, 72 patients taking the drug remained.

All the participants had depressed left ventricular ejection fraction (< 40%) as per the study protocol. Patients were interviewed about any cardiovascular drug use, and NYHA functional class was determined. Systolic and diastolic blood pressure and heart rates were measured at the beginning of the study and in the third month. Laboratory parameters including serum electrolytes were assayed. Nebivolol 5 mg/day was started in all patients and the same dose was continued throughout the study period.

Patients with β-blocker use in the previous four weeks, electrolyte abnormalities, any drug use prolonging QT interval, coronary artery disease or acute coronary syndromes, pacemakers, atrial fibrillation, and significant hepatic and renal dysfunction were excluded from the study.

The electrocardiograms (ECG) were recorded after 10 minutes of rest in the supine position, using 12 simultaneously recording leads: three standard (DI, DII, DIII), three unipolar (aVR, aVL, aVF) and six precordial (V1–V6), at a paper speed of 25 mm/s. Readings were done manually by the same two cardiologists blinded to the study. There is evidence that manual measurement is superior to automatic measurement of QTd.^9^,^10^

The QT interval was measured in each lead from the onset of the QRS complex: the beginning of the Q wave to the terminal inscription of the T wave in the lead with clearly identified T-wave termination, or from the beginning of the R wave if the Q wave was absent. The terminal inscription of the T wave was determined as the return to the TP baseline. When a U wave was present, the QT interval was measured to the nadir of the curve between the T and U waves. The QTcd was defined as the difference between the maximum and minimum of the QTc intervals, measured in milliseconds in any of the measured ECG leads.

## Statistical analysis

Data were analysed with SPSS software version 15.0 for Windows (SPSS Inc, Chicago, Illinois, USA). Continuous variables are presented as mean ± SD and categorical variables as frequency and percentage. The Kolmogorov–Smirnov test was applied to assess the distribution of continuous variables. The Student’s *t*-test was used to compare normally distributed continuous variables and the Mann–Whitney U-test for variables without normal distribution. Pearson correlation was used for the correlation analysis. The mean inter-observer difference and inter-class coefficients were used to evaluate inter-observer variability. A two-tailed *p*-value < 0.05 was considered statistically significant.

## Results

Baseline characteristics of patients including demographic, clinical and laboratory parameters are outlined in Table 1. The study population was mostly elderly patients and 69.4% were over 65 years. Approximately two-thirds of the patients were white males. All patients were on a drug affecting the renin–angiotensin–aldosteron system. NYHA functional class II was the most common category in the population. No discontinuation of the study drug was observed during the study period.

**Table 1:** Baseline Demographic, Clinical And Laboratory Characteristics Of The Study Population

*Characteristic*	*Value (n = 72)*
Age (years)	71.0 ± 10.4
Male (%)	45 (62.5)
Diabetes mellitus (%)	6 (8.3)
Smoking (%)	13 (18.1)
Hypertension (%)	23 (31.9)
CCB use (%)	5 (6.9)
ACE inhibitor use (%)	62 (86.1)
ARB use (%)	10 (13.9)
Statin use (%)	10 (13.9)
Aldosterone antagonist use (%)	21 (29.2)
Diuretic use (%)	68 (94.4)
Digoxin use (%)	16 (22.2)
NYHA class	
I (%)	4 (5.6)
II (%)	47 (65.3)
III (%)	19 (26.4)
IV (%)	2 (2.8)
LVEF (%)	32.3 ± 5.0
Creatinine (mg/dl)	0.96 ± 0.27
Na (mmol/l)	136.3 ± 4.2
K (mmol/l)	4.2 ± 0.6
Ca (mmol/l)	2.37 ± 0.12

ACE: angiotensin converting enzyme; ARB: angiotensin receptor blocker; CCB: calcium channel blocker; LVEF: left ventricular ejection fraction; NYHA: New York Heart Association.

At the end of the study, vital parameters of patients including systolic (110 ± 15 vs 100 ± 15 mmHg, *p* < 0.001) and diastolic (72 ± 10 vs 68 ± 10 mmHg, *p* < 0.001) blood pressure and heart rates (84 ± 11 vs 74 ± 11 bpm, *p* < 0.001) were significantly decreased, as expected. However, there were no significant changes in the left ventricular ejection fraction and NYHA functional class of the patients between the baseline and follow-up values at one and three months (32.3 ± 5.0 vs 32.4 ± 4.9%, *p* = 0.327 and 2.3 ± 0.6 vs 2.2 ± 0.5%, *p* = 0.103), respectively.

QTc and QTcd were measured at baseline and in the first and third month of the study. The measurements and calculations were performed by two independent observers blinded to the study protocol. The mean values of the QT dynamics from two independent observers are given in Table 2. In the whole population, both QTc and QTcd were significantly decreased in the first and third months compared to baseline values (Table 2). QTc and QTcd values correlated significantly between the two observers. Inter-class coefficients and mean inter-observer differences at baseline and in the first and third months are presented in Table 3.

**Table 2:** QT Dynamics In The Whole Population

*Characteristic*	*Baseline*	*First month*	*Third month*
QTc (ms)	455.3 ± 26.7	441.2 ± 25.7*	436.0 ± 28.7*
QTcd (ms)	65.6 ± 5.3	58.2 ± 5.6**	56.0 ± 6.2*

The mean values of two independent observers are presented. *p < 0.001 for baseline to first month and baseline to third month. **p = 0.001 for baseline to first month.

**Table 3:** Inter-Observer Differences And Inter-Class Coefficients Between Two Observers For QT Dynamics

*Characteristics*	*Mean inter-observer difference (ms)*	*Range (ms)*	*Inter-class coefficient*	*95% CI*	p-*value*
QTc baseline	–2.8	–15 to +20	0.986	0.978–0.991	< 0.001
QTcd baseline	–0.5	–2.5 to +3.5	0.999	0.999–1.000	< 0.001
QTc month 1	–3.0	–20 to +20	0.995	0.993–0.997	< 0.001
QTcd month 1	–0.5	–2.5 to +3.5	0.999	0.999–1.000	< 0.001
QTc month 3	–2.6	–15 to +20	0.993	0.988–0.995	< 0.001
QTcd month 3	–0.5	–3.0 to +3.5	0.999	0.999–1.000	< 0.001

In male subjects, both QTc and QTcd were significantly decreased in the first and third months compared to baseline values. In female subjects, both QTc and QTcd were significantly decreased in the third month compared to baseline values. However, in the first month of follow up, no significant decrease was detected in QTc or QTcd compared to baseline values. In addition, both QTc and QTcd were significantly decreased in the third month compared to the first month (data not presented).

There was a significant positive correlation between age and baseline QTcd in the whole study population (β = 0.567, *p* < 0.001). There was no significant difference between patients in NHYA class I–II and III–IV, according to QTc and QTcd at baseline and in the first and third months (*p* > 0.05 for all).

## Discussion

We found that nebivolol therapy significantly improved both QTc and QTcd parameters in patients with systolic heart failure, which has not been reported in the literature before. Sympathetic tone, excitation–contraction coupling and myocardial fibrosis may be the reasons for impaired QT dynamics in heart failure. Both QTc and QTcd are the indicators of the heterogeneity of myocardial repolarisation and electrical instability.^8^,^11^ The action potential is prolonged and repolarisation is delayed in heart failure patients.

The QT interval on the surface ECG is a readily measurable reflection of cardiac repolarisation. The QT interval is an index of ventricular repolarisation that is directly influenced by myocardial health and autonomic nervous system activity. Patients with heart failure and prolonged action potential durations have abnormalities of the QT interval. In a small group of heart failure patients, QT dispersion has been shown to be a marker of electrical instability and increased risk of sudden death.^12^ In patients with known repolarisation abnormalities, the QTcd has been demonstrated to be a better prognostic indicator of arrhythmic risk than the QT itself.^13^ Unstable ventricular repolarisation may contribute to the development of ventricular tachycardia and fibrillation.

It is well known that β-blockers are the only anti-arrhythmic drug class effectively reducing mortality and arrhythmic sudden death in patients with heart failure.^14^ These drugs have been also shown to improve QT dynamics.^15^-^17^

It has been demonstrated that nebivolol therapy reduced the composite risk of all-cause mortality or cardiovascular hospital admission compared with placebo in patients with heart failure.^4^ Nebivolol therapy as an antihypertensive drug has been extensively studied and approved. Its effect on QT dynamics in hypertensive patients has shown that nebivolol significantly reduced QTcd in hypertensive subjects without affecting left ventricular mass.^7^ In our study, no statistically significant difference was found between hypertensive and non-hypertensive patients according to QTc and QTcd at baseline and in the first and third months of follow up (*p* > 0.05 for all).

Statin therapy has also been found to be associated with improved QT dynamics in heart failure patients.^18^ In our study there was no significant difference between patients on statin therapy and those without statins, according to QTc and QTcd parameters at baseline (*p* > 0.05 for all). We found that in female subjects, neither QT dynamic significantly decreased in the first month compared to baseline. The reason for this could be the small number of females, which did not reach statistical significance.

Although ACE inhibitor therapy has been shown to decrease QTd,^19^ in our study no significant difference was found between patients on ACE inhibitors and those not on them at baseline (*p* > 0.05). Also, no significant difference was found between those on digoxin and those not on digoxin at baseline (*p* > 0.05).

Neither QTc nor QTcd were different among NHYA functional classes at baseline and in the first and third months of follow up. This demonstrates that QT dynamics were impaired regardless of NYHA functional class. Since there were no significant changes in the left ventricular ejection fraction and NYHA functional class, this indicates that the QT changes were the result of the drug’s effect on the heart rate and blood pressure and not due to improvement in the heart failure.

A limitation of this study was that this was a case–control observational study without primary end-points such as hospitalisation for heart failure, cardiac and total mortality. Therefore, randomised blinded studies with longer follow-up periods are needed. In addition, we only studied heart failure patients with systolic dysfunction and normal coronary arteries. Similar studies should be performed in heart failure patients with preserved systolic function and coronary heart disease.

## Conclusion

Similar to other older-generation β-blockers, nebivolol is a selective β_1_-blocker with vasodilator properties that have the potential to improve QT dynamics in patients with heart failure. Therefore the good clinical results obtained in the heart failure patients in our study can partly be attributed to improvement in their QT dynamics.
